# Cost-effectiveness of serplulimab as first-line therapy for extensive-stage small cell lung cancer in China

**DOI:** 10.3389/fimmu.2023.1223020

**Published:** 2023-08-31

**Authors:** Guiyuan Xiang, Tingting Jiang, Lanlan Gan, Yuanlin Wu, Ni Zhang, Haiyan Xing, Hui Su, Yanping Li, Dan Peng, Rui Ni, Yao Liu

**Affiliations:** Department of Pharmacy, Daping Hospital, Army Medical University, Chongqing, China

**Keywords:** cost-effectiveness, serplulimab, chemotherapy, extensive-stage small cell lung cancer, first-line treatment

## Abstract

**Objective:**

The ASTRUM-005 trial demonstrated that adding serplulimab to chemotherapy significantly prolonged the survival of patients with extensive-stage small cell lung cancer (SCLC), but also increased the risk of adverse events. Given the high cost of serplulimab compared to chemotherapy, this study aimed to evaluate the cost-effectiveness of serplulimab plus chemotherapy as a first-line treatment for extensive-stage SCLC from the perspective of China’s healthcare system.

**Methods:**

A Markov model was developed to simulate the disease process of extensive-stage SCLC and estimate the health outcomes and direct medical costs of patients. Scenario analyses, univariate sensitivity analyses, and probabilistic sensitivity analyses were conducted to explore the impact of different parameters on model uncertainty. The primary model outcomes included costs, life-years (LYs), quality-adjusted life-years (QALYs), and the incremental cost-effectiveness ratio (ICER).

**Results:**

Compared to placebo plus chemotherapy, serplulimab plus chemotherapy resulted in an additional 0.25 life-years and 0.15 QALYs, but also increased costs by $26,402, resulting in an ICER of 179,161 USD/QALY. Sensitivity analysis showed that the ICER was most sensitive to the cost of serplulimab, and the probability that serplulimab was cost-effective when added to chemotherapy was only 0 at the willingness-to-pay threshold of 37,423 USD/QALY. Scenario analysis revealed that price discounts on serplulimab could increase its probability of being cost-effective.

**Conclusion:**

Serplulimab plus chemotherapy is not a cost-effective strategy for first-line treatment of extensive-stage SCLC in China. Price discounts on serplulimab can enhance its cost-effectiveness.

## 1 Introduction

Lung cancer is the leading cause of cancer-related deaths worldwide, with a staggering 2.2 million new cases and 1.8 million deaths annually (1). In China, lung cancer has the highest incidence and mortality rate among all cancers, with approximately 870,000 new cases and 770,000 deaths each year (2). Non-small cell lung cancer (NSCLC) and small cell lung cancer (SCLC) are the two main histological classifications of lung cancer, with SCLC accounting for 10-15% of cases and having a 5-year survival rate of less than 7% (3). SCLC can be further divided into limited and extensive stages, with extensive-stage accounting for about 65% of new cases (4, 5). Platinum-based chemotherapy, particularly etoposide plus platinum (carboplatin/cisplatin), has been the standard first-line treatment for extensive-stage SCLC for the past three decades (6). However, in recent years, immunotherapy, including immune checkpoint inhibitors (ICIs), has emerged as a promising treatment option for SCLC. Several studies have shown that the addition of ICIs to chemotherapy for extensive-stage SCLC provides longer survival than chemotherapy alone, but some ICIs have been associated with increased incidence of serious adverse events (7–11).

Serplulimab, a fully humanized immunoglobulin G4 (IgG4) monoclonal antibody targeting the programmed cell death protein 1 (PD-1) receptor, was approved by the National Medical Products Administration (NMPA) in January 2023 for first-line treatment of extensive-stage SCLC based on the results of the ASTRUM-005 trial. ASTRUM-005 (ClinicalTrials.gov Identifier: NCT04063163) was a multicenter, randomized phase III trial compared serplulimab plus chemotherapy with placebo plus chemotherapy in the first-line treatment for patients with extensive-stage SCLC. The trial demonstrated that serplulimab plus chemotherapy prolonged median overall survival (OS) by 4.5 months (15.4 months vs. 10.9 months; Hazard ratio, 0.63 [95%CI, 0.49-0.82]) and median progression-free survival (PFS) by 1.4 months (5.7 months vs. 4.3 months; Hazard ratio, 0.48 [95%CI, 0.38-0.59]) compared with placebo plus chemotherapy. However, the serplulimab plus chemotherapy group had a higher incidence of grade 3 or higher treatment-related adverse events than the placebo group (33.2% vs. 27.6%) (12).

Despite the promising results of serplulimab, its cost-effectiveness needs to be evaluated. With the increasing number of newly marketed therapeutic drugs, clinical treatment must consider patients’ affordability and health insurance funds’ sustainability. Therefore, this study aims to evaluate the cost-effectiveness of serplulimab in combination with chemotherapy as first-line therapy for patients with extensive-stage SCLC from the perspective of China’s healthcare system.

## 2 Methods

### 2.1 Model overview

**Figure 1:**
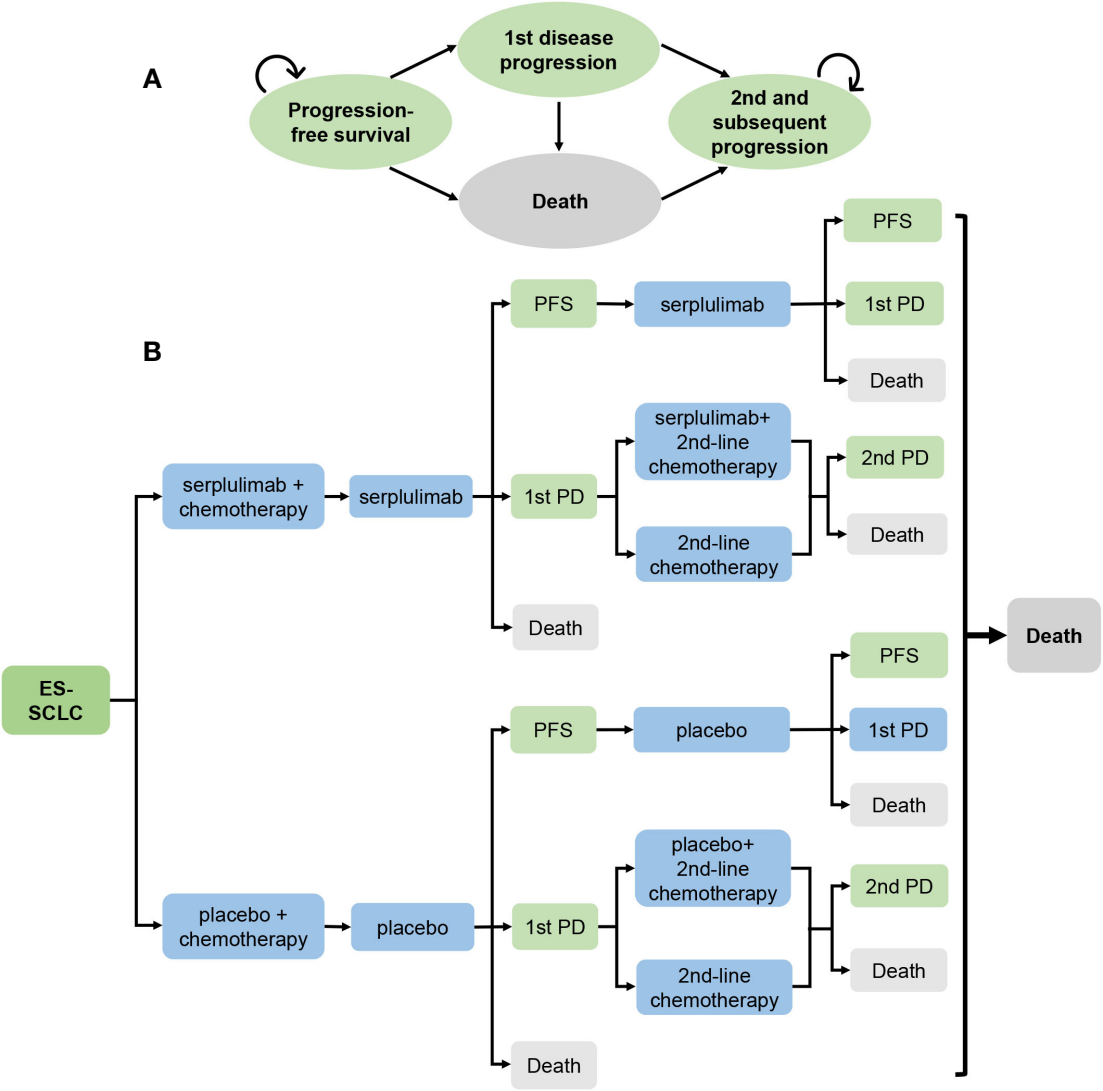
Model diagrams for analysis comparing serplulimab treatment vs placebo treatment in extensive-stage small cell lung cancer. **(A)** Simplified Markov Model. **(B)** Potential treatment pathways and decision tree. ES-SCLC, extensive-stage small cell lung cancer; PD, progressive disease; PFS, Progression-free survival.

This study adhered to the Consolidated Health Economic Evaluation Reporting Standards 2022 (CHEERS 2022) guidelines for health economic evaluation (13). A Markov model was developed to estimate the cost and effectiveness of two treatment groups from the perspective of the healthcare system. The model consisted of four health states: progression-free survival (PFS), first disease progression (1st PD), second and subsequent disease progression, and death. Patients entered the model in the PFS state and then transitioned to other states (**Figure 1A**). The model cycle was 3 weeks, the time horizon was lifetime, i.e., the model was run until all patients died, yielding an actual time horizon of 7.44 years, and a half-cycle correction was applied in this model.

A total of 585 patients were randomly assigned to the serplulimab and placebo treatment groups in a 2:1 ratio upon entry into the model. The serplulimab and placebo groups received four 21-day cycles of serplulimab (4.5 mg/kg) and placebo treatment, respectively, while both groups received four 21-day cycles of etoposide (100mg/m2 on days 1, 2, and 3) and carboplatin (within the area under the concentration-time curve (AUC) of 5 mg/mL/min on day 1) treatment. The two groups then received serplulimab or placebo monotherapy until the first disease progression or death, respectively. Patients received subsequent treatment with second-line chemotherapy after the first disease progression until the second disease progression or death, and patients who could benefit from serplulimab or placebo in addition to second-line chemotherapy were also treated with serplulimab or placebo until the second disease progression or death, respectively. After the second disease progression, all patients received the best supportive care until death (**Figure 1B**). Patients in the model received second-line chemotherapy with irinotecan plus carboplatin or etoposide plus carboplatin in subsequent treatment. Based on data published in ASTRUM-005, we assumed that 55.23% of patients in the serplulimab group received serplulimab in subsequent treatment, and 50.23% of patients in the placebo group received placebo. Patients received carboplatin rather than cisplatin in subsequent treatment because of its lower toxicity than cisplatin.

### 2.2 Model survival and progression risk estimates

We extracted data from the published PFS and OS survival curves in the ASTRUM-005 trial using webplotdigitizer (Version 4.6, https://automeris.io/WebPlotDigitizer). We then generated pseudo-individual patient data according to Hoyle’s algorithm (14). The survival curves were reconstructed using R software (Version 4.2.2, https://www.r-project.org/) and fitted to various survival distribution models, including Exponential, Weibull, Logistic, Log-logistic, and Log-normal models, to obtain the scale and shape parameters. The survival curves were extrapolated until all patients died. Based on the Akaike information criterion, Bayesian information criterion, and visual inspection, we selected the Weibull distribution model. We calculated the time-varying transition probabilities between each survival state using the survival function S(t) = exp(-λt^γ) (λ > 0; γ > 0). First, we calculated the PFS and OS probabilities at time t, denoted as P(t) and O(t), respectively. Then the probability of disease progression at t is calculated as Prob(FtP) = [(P(t) − P(t + 1)]/P(t), and the probability of PD to death is Prob(PtD) = [(O(t) - O(t + 1)]/[(O(t) - P(t)]. The transition probabilities from PFS state to death state used a natural mortality rate in China in 2021 (15).

### 2.3 Cost estimates

From the healthcare system perspective, the model included direct medical costs such as drug costs, adverse events, best supportive care, follow-up, hospitalization, and laboratory test costs. Drug prices were obtained from the MENET database as the average of the bid prices for drug procurement across the provinces of China (16). Other costs were derived from published literature (17, 18), We adjusted costs for inflation to reflect 2022 price levels using the Chinese consumer price index (19). The exchange rate of USD 1 = CNY 6.87 in January 2023 was used in this study (20). The mean age of the patients in the model was 61.1 years, and the mean weight of the patients was assumed to be 65 kg, with a body surface area of 1.72 m^2^ and a serum creatinine level of 1 mg/dL or 88.4 μmol/L. All doses were rounded to the nearest milligram. Adverse events of grade 3 and above with an incidence of 5% or higher were considered. The adverse events in both groups were decreased neutrophil count, decreased white blood cell count, decreased platelet count, and anemia in descending order incidence (**Table 1**).

**Table 1:** Model inputs.

Parameter	Base case	Range		Distribution	Source
		Low	High		
Treatment cost per cycle ($)					
Serplulimab	2382.65	1906.12	2859.18	Gamma	16
Etoposide	362.36	289.89	434.83	Gamma	16
Irinotecan	312.03	249.62	374.44	Gamma	16
Carboplatin	55.41	44.33	66.49	Gamma	16
Best Supportive care	344.76	275.81	413.71	Gamma	17
Routine follow-up*	87.42	69.94	104.90	Gamma	17
Cost of managing adverse events ($)					
Anemia	533.61	426.89	640.33	Gamma	18
Decreased white blood cell count	489.30	391.44	587.16	Gamma	18
Decreased neutrophil count	88.42	70.73	106.10	Gamma	18
Decreased platelet count	1106.70	885.36	1328.04	Gamma	18
Health utility					
Progression-free survival	0.673	0.5384	0.8076	Beta	21
Progressive disease	0.473	0.3784	0.5676	Beta	21
Health disutility					
Anemia	0.073	0.0584	0.0876	Beta	18
Decreased white blood cell count	0.2	0.16	0.24	Beta	18
Decreased neutrophil count	0.2	0.16	0.24	Beta	21
Decreased platelet count	0.19	0.152	0.228	Beta	21
**Discount rate**	0.05	0	0.08	Fixed	–
**Time horizon(years)**	7.44	2.00	7.44	Fixed	–
Risk of AEs in serplulimab group					
Anemia	0.054	0.0432	0.0648	Beta	12
Decreased white blood cell count	0.085	0.0680	0.1020	Beta	12
Decreased neutrophil count	0.141	0.1128	0.1692	Beta	12
Decreased platelet count	0.062	0.0496	0.0744	Beta	12
Risk of AEs in placebo group					
Anemia	0.056	0.0448	0.0672	Beta	12
Decreased white blood cell count	0.087	0.0696	0.1044	Beta	12
Decreased neutrophil count	0.138	0.1104	0.1656	Beta	12
Decreased platelet count	0.082	0.0656	0.0984	Beta	12

AEs, adverse events; PD, progressive disease; PFS, Progression-free survival.

* The routine follow-up cost included outpatient physician visit, hospitalization, and laboratory tests.

### 2.4 Utility estimates

To measure effectiveness in this study, quality-adjusted life-years (QALYs) and life-years (LYs) were used. Since ASTRUM-005 did not report quality of life data, health utility values were obtained from published literature (18, 21). We used a utility of 0.673 in the PFS state and 0.473 in the first and subsequent disease progression states, and 0 in the death state for both treatment groups. The disutilities applied to adverse events were as follows: decreased neutrophil count (-0.2), decreased white blood cell count (-0.2), decreased platelet count (-0.19), and anemia (-0.073). In accordance with the recommendations of the Chinese Pharmacoeconomic Evaluation Guidelines (22), a discount rate of 5% per year was applied to both health outcomes and costs in both treatment groups in this study (**Table 1**).

### 2.5 Sensitivity analysis and scenario analysis

We conducted univariate sensitivity analysis and probabilistic sensitivity analysis to assess the robustness of the model. In the univariate sensitivity analysis, we varied cost, utility, and probability variables by ±20% of the baseline value, while the discount rate had a baseline value of 5% and was varied from 0-8%, as well as time horizon with a baseline value of 7.44 years, with a range of variation from 2.00-7.44 years. Additionally, we performed probabilistic sensitivity analysis using 1000 Monte Carlo simulations to explore the effect of simultaneous changes in multiple variables on the uncertainty of the model. The variables were assumed to vary in a specific distribution pattern, with cost variables assumed to follow a Gamma distribution, and utility and probability variables assumed to follow a Beta distribution, while the discount rate and time horizon remained fixed. The willingness-to-pay (WTP) threshold in the cost-effectiveness analysis was set at $37,423 per QALY, which is three times the per capita Gross Domestic Product (GDP) of China in 2022 (23). Furthermore, we evaluated the impact of different price discount scenarios for serplulimab on ICER to provide a reference for health insurance reimbursement.

## 3 Results

### 3.1 Base-case results

The base-case results showed that the total cost of treatment per patient in the placebo group was $6789, while the cost in the serplulimab group was $33,191, which is $26,402 more than the placebo group (**Table 2**). In terms of health outcomes, the placebo and serplulimab treatments resulted in 1.25 and 1.51 life-years, respectively. After accounting for quality of life, each patient in the placebo and serplulimab groups gained 0.64 QALYs and 0.79 QALYs, respectively. The serplulimab group gained 0.26 life-years and 0.15 QALYs more than the placebo group per patient. Therefore, the incremental cost-effectiveness ratio (ICER) in the serplulimab group was $179,161/QALY or $102,535/LY compared to the placebo group. At the WTP threshold of 37,423 USD/QALY, the serplulimab treatment is not cost-effective.

**Table 2:** Base case results.

Treatment	Total cost, $	LYs	QALYs	Incremental			ICER ($/QALY)
				Cost, $	LYs	QALYs	
Base case							
Placebo+ chemotherapy	6789	1.25	0.64	NA	NA	NA	NA
Serplulimab+ chemotherapy	33,191	1.51	0.79	26,402	0.26	0.15	179,161
Scenario analysis							
Placebo+ chemotherapy	6789	1.25	0.64	NA	NA	NA	NA
Serplulimab+ chemotherapy	12,304	1.51	0.79	5515	0.26	0.15	37,423

ICER, incremental cost-effectiveness ratio; LYs, life-years; NA, not applicable; QALYs, quality-adjusted life-years.

### 3.2 Sensitivity analysis

**Figure 2:**
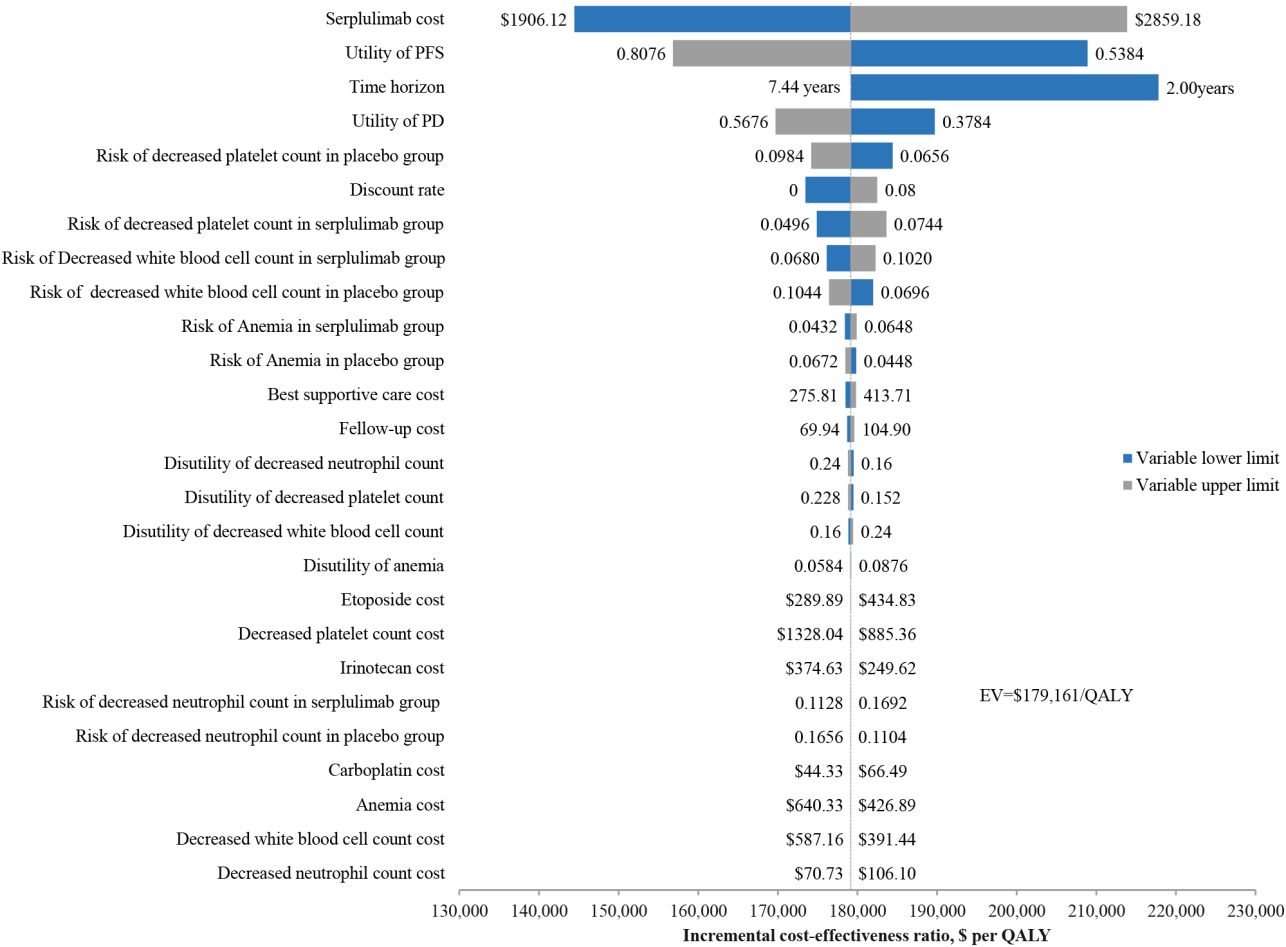
Univariate sensitivity analysis results for the base case analysis. QALY, quality-adjusted life-years.

The results of the univariate sensitivity analysis (**Figure 2**) indicated that the cost of serplulimab had the greatest impact on the ICER, with a range of $144,443-$213,879 per QALY when the cost of serplulimab varied by ±20%. The utility of the PFS state is second only to the cost of serplulimab in terms of its impact on ICER, and is followed by time horizon and the utility of PD state. Moreover, ICER values varied negatively with time horizon. Additionally, the risk of adverse events in the serplulimab and placebo groups also had a significant impact on the ICER. The discount rate had a significant effect on the ICER in the range of 0-8% change. However, regardless of which parameter varied individually within the established range, the ICER value remained above the $37,423 WTP threshold per QALY, indicating that the serplulimab group was consistently not cost-effective compared to the placebo group.

**Figure 3:**
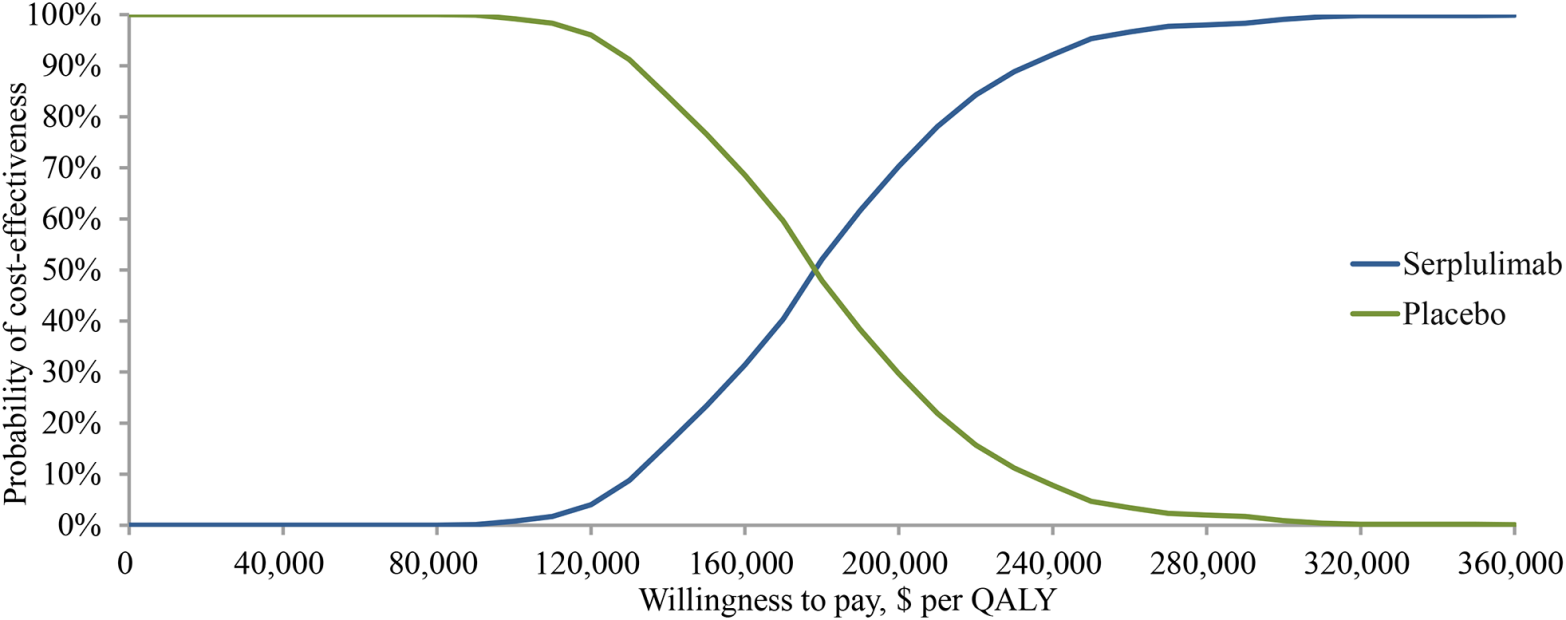
Cost-effectiveness acceptability curve for the base case analysis. QALY, quality-adjusted life-years.

The probabilistic sensitivity analysis showed a mean incremental cost of $26,392, a mean incremental effectiveness of 0.15 QALYs, and an ICER of $178,927 per QALY for serplulimab versus placebo over 1000 Monte Carlo simulation iterations. The cost-effectiveness acceptability curve (**Figure 3**) shows that when the WTP threshold was set at $37,423 per QALY, the probability of serplulimab and placebo groups being cost-effective was 0 and 100%, respectively. The probability of serplulimab being cost-effective increased as the WTP increased, but the ICERs were above the WTP threshold of $37,423 per QALY for most combinations of variables.

### 3.3 Scenario analysis

We performed scenario analysis of different price discounts for serplulimab in the model. The results showed that when the cost of serplulimab decreased by 20% and 50%, the ICER was $144,443/QALY and $92,365/QALY, respectively. Probabilistic sensitivity analysis showed that the probability of cost-effectiveness of serplulimab was 0 in both cases. With other conditions remaining unchanged, the serplulimab group was cost-effective compared to the placebo group only if the price of serplulimab was reduced by 81.65% from the current price. At this price point, the total cost of treatment per patient in the serplulimab group was $12,304, and the ICER was $37,423 per QALY.

## 4 Discussion

The results of the phase III trial ASTRUM-005 revealed the significant efficacy of serplulimab in the treatment of small cell lung cancer, and serplulimab also became the first marketed PD-1 drug for the treatment of extensive-stage small cell lung cancer. We developed a four-state Markov model to evaluate the cost-effectiveness of serplulimab as a first-line treatment for extensive-stage SCLC, based on the results of the ASTRUM-005 trial. Our analysis suggested that the addition of serplulimab to chemotherapy resulted in an average survival benefit of 3.12 months per patient, equivalent to 0.15 QALYs gained. However, the cost of serplulimab was $26,402 per patient, resulting in an ICER of $179,161 per QALY. At the current WTP threshold of $37,423 per QALY in China, serplulimab plus chemotherapy is not considered a cost-effective treatment option.

The sensitivity analysis showed that the probability of adding serplulimab to chemotherapy was 0. The sensitivity analyses suggested that the addition of serplulimab to chemotherapy is less likely to be cost-effective. Since the price of serplulimab has the greatest impact on the model, we also conducted a scenario analysis to evaluate the impact of the cost of serplulimab on the model results. Our findings showed that the price of serplulimab would need to be reduced by at least 81.65% to make the immunotherapy regimen cost-effective at the base-case. Currently, the average price of serplulimab in China is 5588 Chinese yuan per 100 mg ($813.39/100 mg), implying that the price of serplulimab would need to drop to about 1205.31 Chinese yuan per 100 mg ($149.25/100 mg) for serplulimab to be considered cost-effective at the WTP threshold of three times per capita GDP of China. Currently, pharmacoeconomic evaluation plays an important role in the adjustments of the National Reimbursement Drug List (NRDL) of China. The Chinese government has conducted annual NRDL access negotiations since 2016, with the National Healthcare Security Administration (NHSA) as the main management department. In the process of access, firstly, expert review panel conducts a comprehensive evaluation on the safety, effectiveness, economy, innovation, suitability, and accessibility of drugs, and sets the payment price and negotiates base price of drugs based on the results of economic evaluation and budget impact analysis, and then negotiating expert panel conducts price negotiation with drug manufacturers on the basis of the base price to form the final payment price, with the final payment price not exceeding 115% of the base price (24). After a successful negotiation, drug prices would drop dramatically, NHSA will set the range of medical insurance payment indications and formulate a single medical insurance payment price. If the manufacturer of serplulimab succeed in negotiating with the NHSA in the future, serplulimab’s cost-effectiveness would be greatly improved. In addition to the price of serplulimab, the health utility values of PFS and PD states also have a significant impact on the model, but there is currently a lack of research on the quality of life of patients with small cell lung cancer, and it is necessary to carry out relevant studies in the future to fill this gap to promote the economic evaluation of small cell lung cancer treatment drugs in China.

The serplulimab regimen is not cost-effective at the current WTP threshold in China. Furthermore, when the results of this study are extrapolated to other high-income countries, such as the United States with a WTP threshold of $150,000 per QALY and the United Kingdom with a WTP threshold of £50,000 per QALY (25, 26), the combination of serplulimab and chemotherapy is still not considered cost-effective. However, if a higher WTP threshold is set for SCLC patients in the United States (27), this treatment regimen may become cost-effective.

Currently, Immunotherapeutic agents approved for extensive-stage SCLC in China include atezolizumab and durvalumab, in addition to serplulimab. Three studies have analyzed the cost-effectiveness of atezolizumab and durvalumab in combination with carboplatin and etoposide for the first-line treatment of extensive-stage SCLC from a Chinese payer perspective. The results showed that compared to chemotherapy regimens, the ICERs for atezolizumab plus chemotherapy were $489,013 per QALY, and for durvalumab plus chemotherapy, they were $192,591 per QALY and $230,142.9 per QALY (28–30). Therefore, neither combination regimen was cost-effective compared to chemotherapy regimens. Although the ICER for serplulimab plus chemotherapy in this study was lower than those for atezolizumab and durvalumab, it was still well above the WTP threshold. Reducing the price of serplulimab remains a potential solution to make it cost-effective. Meanwhile, risk-sharing agreements are still a worthwhile payment method for China’s health insurance administration. Serplulimab was only recently approved for the first-line treatment of extensive-stage SCLC in 2023, and further evaluation of its economics based on real-world data generated in clinical use is still needed to inform clinical and health insurance reimbursement decisions. While value-based pricing has been successfully applied in China, the increase in indications for serplulimab or other ICIs, and the varying clinical value of different indications, make multi-indication pricing another promising approach. Therefore, conducting an economic evaluation of multi-indications in the future is necessary to provide a reference for the authorities.

In terms of clinical treatment decision-making for extensive-stage SCLC, we suggest that clinicians need to consider not only the patient’s disease status, but also the patient’s ability and willingness to pay when developing a treatment plan, and prioritize the selection of medicines that have been included in the NRDL. Standard chemotherapy may be preferred for patients with poor ability to pay but who tolerate platinum-based agents well. At the same time, China’s current economic development is highly uneven, and the level of economic development among provinces is quite different. Different regions should combine the local economic development level when referring to the results of this study. For example, the per capita regional GDP of Beijing and Shanghai in 2022 (27,729 USD in Beijing and 26,252 USD in Shanghai) is more than twice the national per capita GDP, the probability of serplulimab being cost-effective in this location is increased (31, 32). In addition, it is recommended that the manufacturer of serplulimab develop a suitable Patient Assistance Program (PAP) to provide pharmaceutical assistance to patients who are impoverished due to illness or who are enduring catastrophic health expenditures, in order to improve the accessibility of serplulimab.

There are still several limitations in our study. ASTRUM-005 reported a large number of second-line treatment regimens during the maintenance phase, which led us to simplify the model by assuming that the second-line chemotherapy regimen is only irinotecan plus carboplatin or etoposide plus carboplatin. We did not consider targeted therapies, herbal or traditional Chinese medicine, or other immunotherapies except serplulimab and placebo, which may have caused ICER bias. Additionally, since ASTRUM-005 has no published quality of life measurement data, and there is no study on the health utility of SCLC in China, the health state utility values in this study were mainly derived from the study of health state utilities of NSCLC by Nafees B et al. However, the malignancy of SCLC is higher than that of NSCLC, and its actual utility may be lower than that of NSCLC, resulting in a higher ICER.

## 5 Conclusion

In conclusion, this study found that serplulimab plus chemotherapy is not a cost-effective treatment strategy for extensive-stage SCLC in China at the current WTP threshold. Reducing the price of serplulimab remains a potential solution to make it cost-effective. Further evaluation of its economics based on real-world data generated in clinical use is still needed to inform clinical and health insurance reimbursement decisions.

## Data availability statement

The original contributions presented in the study are included in the article/supplementary material. Further inquiries can be directed to the corresponding author.

## Author contributions

YLiu and GX were responsible for the conception and design of the study. GX contributed to the model building and drafted the manuscript. YLiu supervised the project. All authors contributed to the article and approved the submitted version.
